# Book Reviews

**Published:** 1876-01

**Authors:** 


### Book Reviews.

[NOTE.—All works reviewed in the pages of the CHICAGO MEDICAL JOURNAL AND EXAMINER may be found in the extensive stock of W. B. KEEN, COOKE & Co., whose catalogue of Medical Books will be sent to any address upon request.]

THE UNITED STATES GOVERNMENT REPORT ON CHOLERA.

I. CHOLERA EPIDEMIC OF 1873 IN THE UNITED STATES. The Introduction of Epidemic Cholera through the Agency of the Mercantile Marine: Suggestions of Measures of Prevention. By *John M. Woodworth, M.D.*, Supervising Surgeon U. S. (Merchant) Marine Hospital Service. Washington: 1875. 28 Pages.

II. REPORTS PREPARED UNDER THE DIRECTION OF THE SURGEON-GENERAL OF THE ARMY. (*a.*)—History of the Cholera Epidemic of 1873 in the United States. By *Ely McClellan, M.D.*, Assistant Surgeon U. S. A. 513 Pages. (*b.*)—History of the Travels of Asiatic Cholera. By *John C. Peters, M.D.*, of New York City, and *Ely McClellan, M.D.*, Assistant Surgeon U. S. A. 192 Pages. (*c.*)—Bibliography of Cholera. By *John S. Billings, M.D.*, Assistant Surgeon U. S. A. 316 Pages. Washington: Government Printing Office. 1875.

Observant members of the profession, will not soon forget their surprise and gratification at the appearance of the *Medical and Surgical History of the War of the Rebellion*, and at the way in which it was received by the most competent judges abroad. No such contribution to medicine, gathered from the medical and surgical incidents and accidents of a great war, has probably ever been made before or since. The approbation with which these labors of the Medical Department of the United States Army were received, has not been without influence in stimulating labors like the present, contained in a ponderous octavo of over 1,000 pages. Such a work could have been accomplished in so short a period of time, only through the almost unrivaled opportunities for collecting information, in reference to such a subject, as is at the disposal of the Medical Department of the Army.

It can hardly be said to be an *opus magnum*, with such remarkable resources, literary and original, at command. Still it is such a work as could hardly have been executed from private hands. In either or both of two ways it may be valuable, either on account of the nature and range of the materials, or the character of the discussion to which they are subjected. The former may be good, and the latter inadequate, or even bad. Upon these two points we will concentrate, though not formally, our attention.

The first part is on "The Introduction of Epidemic Cholera, through the Agency of the Mercantile Marine: Suggestions of Measures of Prevention," and is by the efficient and accomplished Supervising Surgeon of the United States Merchant Marine Hospital Service, Dr. John M. Woodworth. This paper of 28 pages, is only one of the many evidences he has given of an enlightened activity, in improving his opportunities, to turn his position to account, not only to the service over which he presides, but to the advancement of the best interests of medicine. As the title of his paper indicates, it is devoted principally to a history of the modes of introduction of cholera into the United States, through the Merchant Marine. In respect to the importance of this question, Dr. Woodworth truly says:

"If it be true that 'cholera has always been brought to America by ships,' the task of preventing future outbreaks within our borders, is a problem in preventive medicine the solution of which would seem comparatively easy. Such solution depends, however, in common with the answer to any question, upon the extent and accuracy of our knowledge of the factors of the problem. Hence it will be necessary to state compendiously what is known and accepted concerning the cause of malignant cholera —its origin, character, mode of propagation, transportation, etc.; a summary which will be of additional use in assisting to a correct estimate of the value of any suggested means of prevention or limitation."

Dr. Woodworth does not attempt a full discussion of the subject of his paper, but gives rather a summary of what is considered probable by those most competent to judge, as regards the nature of the cause, and of the modes of its propagation and action. Inasmuch as these are important practical questions, we will quote them at length for the benefit of our readers who may not have seen the work itself. They are as follows:

"I. Malignant cholera is caused by the access of a specific organic poison to the alimentary canal; which poison is developed spontaneously only in certain parts of India, (Hindostan.)

"II. This poison is contained primarily, so far as the world outside of Hindostan is concerned, in the ejections —vomit, stools, and urine—of a person already infected with the disease.

"III. To set up anew the action of the poison, a certain period of incubation with the presence of alkaline moisture is required, which period is completed within one to three days; a temperature favoring decomposition, and moisture or fluid of decided alkaline reaction hastening the process, the reverse retarding.

"IV. Favorable conditions for the growth of the poison are found (1) in ordinary potable water, containing nitrogenous organic impurities, alkaline carbonates, etc.; (2) in decomposing animal and vegetable matter possessing an alkaline reaction; (3) in the alkaline contents of the intestinal portion of the alimentary canal.

"V. The period of morbific activity of the poison—which lasts, under favorable conditions, about three days for a given crop—is characterized by the presence of bacteria, which appear at the end of the period of incubation, and disappear at the end of the period of morbific activity. That is to say, a cholera-ejection, or material containing such, is harmless both before the appearance and after the disappearance of bacteria, but is actively poisonous during their presence.

"NOTE.—It is not meant by this that the bacteria so found are the cholera-poison, since they differ in no appreciable manner from bacteria found in a variety of other fluids. Lebert hints that the bacteria may even be the destroyers of the poison.

"VI. The morbific properties of the poison may be preserved in posse for an indefinite period in cholera-ejections dried during the period of incubation, or of infection-matter dried during the period of activity.

"VII. The dried particles of cholera-poison may be carried (in clothing, bedding, etc.) to any distance; and when liberated may find their way direct to the alimentary canal through the medium of the air—by entering the mouth and nose and being swallowed with the saliva—or, less directly, through the medium of water or food in which they have lodged.

"VIII. The poison is destroyed naturally either by the process of growth or by contact with acids: (1) those contained in water or soil; (2) acid-gases in the atmosphere; (3) the acid secretion of the stomach.

"IX. It may also be destroyed artificially (1) by treating the cholera-ejections, or material containing them, with acids; (2) by such acid (gaseous) treatment of contaminated atmosphere; (3) by establishing an acid diathesis of the system in one who has received the poison."

These conclusions if supported by the facts, even though they are provisional, are of great value, and deserve thoughtful attention. We can only give them as they stand, reserving comment until a later part of this paper.

It will not be possible for us to notice at length, the cases in which the transportation of the cholera-poison from one port to another, has been ascertained beyond reasonable doubt, especially in the clothes and other personal effects of patients who have suffered or died from the disease. They are convincing and complete. There can be no doubt that the disease is caused by some material organic agent capable of being conveyed from point to point. Apart from other interesting relations, which such facts suggest, it is to be noted that they have an especial bearing on that hitherto neglected branch of medicine called *preventive*, which is destined to have an increasing importance as time passes by, and as we approach that millenium in medicine when it will be the principal aim, and practical work of physicians to *prevent* rather than *cure* disease. Who can estimate the value of the lives that will be spared and the general suffering that will be prevented, when we shall so understand the cause of cholera, and its mode of action, as in a rational way to arrest its spread, or to render its cause inoperative! Works like this great Government Report, which is so largely given up to the history of the introduction and spread of the disease in our own country, as well as in other parts of the world, are of inestimable value, if the task of criticising and serving up the matter contained is creditably executed. The position of Dr. Woodworth, as the Chief Surgeon to the Merchant Marine Hospital Service, is peculiarly favorable for collecting reliable information on these important questions, for it must of necessity be, that it is through this branch of our Marine rather than that of the organized Naval Service, that the introduction of the cholera from foreign countries must be accomplished. Dr. Woodworth has done his part of this work in a clear and creditable manner.

He enters also on a consideration of the means for preventing the action of the poison, in the case of those who are subject to its influence. Let the following quotations speak for themselves, on this point, and on the prevention of cholera in general:

"It may be alleged that in the foregoing pages too great stress has been laid upon the *acid prophylaxis of cholera*, to the exclusion of all others. But the cumulative evidence of the experience of the last sixty years warrants the ground here taken. Beginning with the year 1814, the cholera-literature down to the present time abounds in proofs, clinical, physiological, pathological, and meteorological, of the efficacy of sulphuric acid, and there can be little doubt, despite the *dicta* of the last International Sanitary Conference, that *we possess in the mineral acids a certain means of prophylaxis against cholera.*"

"It is safe to say that malignant cholera can be excluded from our shores with reasonable certainty through an intelligent sanitary surpervision of the mercantile marine; in which supervision, while the General Government on the one hand, in exercising its delegated powers for the protection and promotion of the general welfare, shall simply acquire and furnish the necessary information; on the other, the ports themselves, thus forewarned and advised, shall be left to enforce the necessary precautionary and preventive measures in accordance with their own local conditions and requirements. For nothing is more clearly proved by the history of cholera than that epidemics of this dreaded disease can be controlled by *vigorous hygienic measures. The true remedy against cholera is preventive medicine.*"

The part of the work which succeeds that of Dr. Woodworth is comprised in twenty-three chapters, as follows: I. Clinical History of the Epidemic of 1873; II. Etiology of the Epidemic of 1873; III. On the Prevention of Cholera; IV. On the Origin of the Epidemic of Cholera that reached the United States in 1873; while chapters V. to XXI. inclusive, are devoted to Narratives of the Epidemics for the same year, in the following States: Louisiana, Mississippi, Arkansas, Tennessee, Illinois, Missouri, Kentucky, Ohio, Indiana, Alabama, West Virginia, Georgia, Minnesota, Pennsylvania, Texas, Iowa and Dakota; while the remaining two chapters contain accounts of "Cholera Cases in New York Harbor," and the "Epidemic (1873) as it affected the United States Army." This portion of the work comprises about 500 pages.

As regards the first chapter, valuable as it may be, as a relation of the symptoms and appearances of cholera, for the purposes of identification and comparison, it does not, so far as we can see, contain anything of importance that is new or valuable when compared with the clinical histories of prior epidemics, that calls for particular remark. Neither will it be possible to enter on a consideration of the accounts, often brief, and not always satisfactory, which have been collected from different physicians residing in the States already named, and whose statements make up the "narratives." Amongst much other matter that is clearly irrelevant they contain many useful facts. This, by far the largest part of the present volume, has a certain value—scientific and historical—and we are glad to see so many fragmentary communications, from so many localities, and from such an extent of territory, preserved in a permanent form.

Neither will it be possible or even profitable, for us to enter into an examination at present, of the trustworthiness and real bearings of the multitudinous details, contained in the second part of the work, which contains a pretty full sketch of the history of the disease from the earliest times down to the present, by Dr. Peters, of New York, and Dr. McClellan, of the United States Army; the latter being the principal author of the volume. But we can commend this to our readers, as one of the fullest in the English language, and in the highest degree valuable and interesting.

Neither will it be possible for us to do more than mention the ponderous bibliography of cholera literature, contributed by Dr. Billings. It is the result of great labor; and however differences in judgment may arise, as to the scheme of classification adopted, it must be acknowledged as an inestimable boon to all students of the literature of this remarkable disease.

We are glad to see such evidences of a wider range in literary activity than has been apparent, until recently, amongst especially English writers in medicine.

This Bibliographical Index deserves to be published separately.

The remainder of our notice will be confined almost exclusively to the second, third and fourth chapters—especially the second, on the "Etiology of the Epidemic of 1873." In this portion of the work its chief interest centres. The point then before us, is the cause of cholera and its *modus operandi.*

As regards the *nature* of the cause, we have the following proposition:

"That Asiatic cholera is an infectious disease resulting from an organic poison, which, gaining entrance into the alimentary canal, acts primarily upon and destroys the intestinal epithelium."

As to the details respecting this "organic poison," there are copious quotations from Macnamara's, Thudicum's and other works on cholera; and with no very definite results. But the object that appeared to Macnamara of most peculiar interest in examining microscopically the discharges and the alimentary mucous membranes, *post mortem*, of cholera patients, was what he calls "molecular matter." It consists of fine, dark granules, or molecules, and pervades in varying degrees, the discharges and the epithelial cells of the diseased mucous membrane. It would be instructive to quote his interesting description of this matter, but we have no space in which to do this. He evidently considers it, rather than the associated bacteria, as the true *materies morbi* of cholera. He says:

"It is from this molecular matter in the vibrionic stage of decomposition, and not from the vibriones themselves, that the dejecta of cholera patients are capable of setting up a morbid action in the intestinal canal of those who may receive it." "The vibriones are but a manifestation of the changes going on in the organic matter, which, when it has passed through the form of vibriones, appears to lose its terrible property of inducing cholera." "The perfectly fresh dejecta in the active stages of the disease contain no vibriones, but toward the end of collapse, when the evacuations are passed less frequently, probably remaining in the intestines for some hours, vibriones may be seen in the fluid immediately after it has been passed."

As regards its nature, he says:

"I have examined these molecules for hours together, with, probably, the highest magnifying power yet constructed, (the $\frac{1}{70}$ of an inch,) in order to bring all the resources of the microscope to bear on this point. I confess I have learned but little through the aid afforded me by this marvelous piece of optical work. The molecular matter, when examined by it, is still nothing more, apparently, than molecular matter—small specks in the epithelial cells. I conceive, however, that the higher the power used, and the more careful the search instituted, the more certain it becomes that this molecular matter is formed in and at the expense of the epithelial cells, blood-globules, gland-cells, or, in fact, any organic matter brought within its influence."

The later statements in relation to the same subject, quoted from an article in the *Centralblatt f. d. Med. Wissenschaften* for January 15, 1874, by Högyes, of Pesth, is not inconsistent with the foregoing.

Let us then suppose for the moment, that the dark "molecular matter" described by Macnamara, is *the* peculiar material product and—in turn—cause of the disease, what is said as to its mode of operation? It appears, especially from the experiments of Högyes, that this matter may act, not only when introduced into the alimentary canal, but also when injected into the veins, and thus made to mingle directly with the blood. What is the mechanism and rationale of the action of the poison?

This is an important question, and one to which we would naturally look for an answer in a ponderous volume like the present. What does the "Report" contain on this topic? Under this relation the elaborate report of Dr. Danforth, of Chicago, upon the pathological specimens obtained by Dr. Hyde in a patient who died of cholera, is the most valuable in the work. In it he makes no distinct mention of the "molecular matter," upon which so much stress was laid by Macnamara. He could find no forms in the discharges nor on the alimentary mucous membrane which he could regard as sufficiently peculiar to be called cholera germs.

And here we notice a great mistake into which the authors of this volume have fallen. The researches of Dr. Danforth are represented as having been made by the Chicago Health Department, and at the instance of Dr. B. C. Miller, sanitary superintendent, when the facts are, that they were made by a committee of the Chicago Society of Physicians and Surgeons, and largely by Dr. J. N. Hyde of the same society, acting with the committee. It is difficult to see how the author could have fallen into such an error as the one to which we allude.

The most important part of Dr. Danforth's report, consists in the account that is given of the appearances presented by the intestinal mucous membrane. The following quotations may suffice to give an idea of them. He says (p. 38):

"Under a power of about eighty diameters, the following appearances are noted: the mucous and muscular layers seemed to have been much disturbed in their relations, and separated widely apart; between them a very beautiful, loosely-woven web of areolar or connective tissue is seen, sending its delicate filaments across the intervening space, with here and there a little vessel, making its way toward the mucous layer; the latter is unusually thin and unusually smooth on its free surface; not a single perfect villus can be seen, but a few "stumps" of villi are easily made out, as though the missing portion had been rudely torn away. Under a power of 260, the surface of the mucous layer is seen to be almost, in fact *quite* denuded of epithelium, since not a single normal club-shaped cell can be seen. The mucous membrane seems to have passed through some scene of violence, during which its villi have been wrenched from their attachments, and its clothing of epithelium stripped from its surface and carried away. It seems almost beyond belief that a few short hours could have so totally changed the intestinal surface, but every section which I have examined from the specimen of intestine now under consideration, presents precisely the same appearances. Peyer's glands do not seem to be much altered, quite to my surprise. Possibly they are slightly swollen, but not otherwise perceptibly altered. But, after all, this is not so surprising; the storm is too brief to affect tissues beneath the surface to any great extent. It is rather like a terrible tornado, desolating everything within its reach, but limited in its ravages to objects presenting salient points of attack. The submucous connective tissue and the muscular layer are both beautifully displayed, but neither present any evidence of disease, unless the unusual separation of the mucous and muscular layers be regarded as such."

These appearances are referred to in various parts of Dr. Danforth's report, but the above extract may be taken as a fair summary of the more characteristic. There were no other phenomena observed in other parts of the body, that could be called to any marked degree distinctive, and our attention is to be fixed, therefore, on the condition of the alimentary mucous membrane. Now how shall we account for this wholesale, violent removal of the epithelium of the mucous membrane of the bowel, as has been so graphically described by Dr. Danforth? Is it done by some agency acting on the free surface of the mucous membrane, or from beneath, so as in the latter case to *push off* its epithelial layers?

No attempt is made to answer this question, which is a legitimate and a highly important one, either by Dr. Danforth, or by the authors of this volume, unless, indeed, we consider as such the bare statement in Proposition I, already quoted from the body of the work, that the organic poison "acts primarily upon and destroys the intestinal epithelium." It may be replied, that it was no part of the intention of the authors of a volume of 1,000 pages on cholera, to discuss questions relating to the *modus operandi* of its cause. While the reply would be a valid one, yet it would not diminish the regret of every thoughtful reader, that questions of so much moment have been ignored in the present work. Who is prepared to discuss such questions, if not those who have consecrated so much time to their study? And here we recall a remark made in the beginning of this notice, that such a work as the present, may prove valuable in either or both of two ways: it may be valuable as a repository of facts—materials, simply as such; or it may be chiefly valuable on account of the character of the discussion to which they are subjected. In the first respect, a work may be highly reputable, while in the latter, it may be inadequate and imperfect in any degree, though the contrary could hardly be the case.

In relation to the present work, it is highly valuable as a mass of materials, bibliographical, historical, actual, but the discussion is altogether inadequate. The matter has not always been judiciously analyzed, and the process of true reflective generalization, has not been carried to even a respectable extent, and certainly not for want of matter or opportunity. There is a remarkable disparity between the labor of collecting materials, and that of digesting them.

But to return. From a consideration of the phenomena of the disease, (since the nature of the cause and its mode of action is known only by inference,) what can be rationally said as to its real pathology, or of the mechanism, or processes, through which its phenomena come to pass? Is it a local disease limited to the mucous membrane, or one of a more general character?

If of the latter kind, how can we account for the remarkable flux from the mucous membrane, and the appearances which it presents *post mortem*, and which are induced with such astonishing rapidity in many cases? Does the cause, whatever it may be, operate through the blood, or the nervous system, or in both ways?

But not to ask further questions, we will declare that in our judgment cholera is not a local disease, if by that term we are to understand that its primary and peculiar sphere of action is that of the mucous membrane of the bowels, and that it is a disease of a more general character, especially involving the action of the splanchnic nerves on the intra-abdominal circulation, and in a way which it will not be possible now to explain, but which it is our intention soon to draw out at length in a special paper on this subject.

The absence of a discussion of such questions, is one of the principal defects of the present work. But taken altogether, it is one which will prove a credit to American medical literature, and will serve, like its predecessors from the medical department of the army, to increase the respect of our transatlantic brethren for the profession and its doings in this country. J. S. J.

A Treatise on Human Physiology; designed for the use of Students and Practitioners of Medicine. By *Jno. C. Dalton, M.D.*, Professor of Physiology and Hygiene, in the College of Physicians and Surgeons, New York, etc. Sixth Edition, Revised and Enlarged, with 316 Illustrations. Pp. 828. Philadelphia: Henry C. Lea. 1875.

The sixth edition of this work comes to us revised, enlarged and enriched by over forty new illustrations.

That a new edition should be so soon demanded, is a flattering endorsement of the book by the profession, and we may safely predict that this edition will be acknowledged to be better than any of its predecessors. It contains about fifty per cent. more reading matter than before, but a change in the typographical arrangement necessitates for this increase the addition of only about one hundred pages to the volume.

The first change the reader familiar with previous editions will notice, is the adoption of the centigrade system of weights and measures, instead of the old pounds, ounces, feet and inches. This is undoubtedly best adapted to scientific purposes, but, as the old system is so much more familiar to the general reader, why did not our author at least give a table showing the value of grammes and centigrammes, in grains, and corresponding estimates for measures of length and capacity? A table of this kind would not only prove a great convenience to many, but would actually aid in the final adoption of the decimal system, by associating it with that which is in actual use.

The centigrade scale has been also adopted for thermometry, but a ready way is given for changing its degrees to those which correspond on the scale of Fahrenheit, the latter, however, usually appear in the text in brackets.

Having thus the two scales in constant conjunction, we shall soon become accustomed to the new and better system, and be glad to adopt it, to the exclusion of the old.

The chapter on "Proximate Principles" is much enlarged and improved. Proximate principles are now described in five classes instead of three, to correspond more nearly with recent progress in organic chemistry. In the chapter on "Food," as in previous editions, we are surprised to find no mention made of our gaseous food, oxygen—that most important of all. For we can survive but a few minutes without it; and with it we can live for days and even weeks—all other alimentation failing. And as oxygen constitutes about one-fourth by weight, of the food required by the body, it would seem to be worthy of a place among the "substances necessary to sustain the process of nutrition," which is our author's definition of food. The chapter upon "Digestion" is very satisfactory. Figure 34, however, is said to be a lobule of the parotid gland of an infant, injected with mercury. To the student who has access to no other illustrations of the salivary glands, this drawing would convey but little information, and that, we fear, not strictly accurate. Figure 48, illustrating Brünner's glands, would in this case afford a clearer impression.

A strange mistake which crept into the fourth edition, has been corrected in this, relating to the digestion of food in the stomach of the ruminants. When an animal of this order has finished browsing, and the process of rumination commences, the food is regurgitated to the mouth by an inverted action of the muscular walls of the paunch and œsophagus, and slowly masticated. It then descends again along the œsophagus; but the lateral opening, which communicates with the first two stomachs, is now closed by muscular action, and the œsophagus, thus converted into a tubular canal, conducts the masticated mass into the *third* stomach, (not the second, as the fourth edition has it). "The exit from this cavity leads directly into the (fourth) abomasus or rennet, the true digestive stomach."

In the table giving the composition of the gastric juice, we see lactic acid has been discarded, and the term "free acid" substituted, which is explained in the text by saying "it is still uncertain whether the reaction of the gastric juice be due to free hydrochloric or free lactic acid." But no mention is made of phosphoric acid in the acid phosphate of lime as an agent in the production of this acidity, a point so much insisted upon by Blandlot, and noticed also by Flint. It certainly deserves a notice in so full an account as we here have of stomach digestion. The chapters on "Intestinal Digestion" and "Absorption" are improved by several new figures. The chapters upon the "Bile" and the "Glycogenic Function of the Liver" are re-written and very satisfactory. The figures illustrating the spectroscopic appearance of the bile and chlorophyll, from the vegetable kingdom, are exceedingly interesting and suggestive. Our author states in another place, "chlorophyll is of the first importance in vegetable physiology, as it is under the influence of this substance, together with that of solar light, that the inorganic ingredients of the soil and the atmosphere are deoxidized and combined in the form of an organic carbo-hydrate. The process of vegetation proper, that is, the production and accumulation of organic material in the form of starch, sugar, cellulose, woody fibre, and the substance of various vegetable tissues, is inseparably dependent on the presence and action of chlorophyll."

If this be true in the vegetable world, may not the coloring matter of the bile be equally necessary for the formation of the carbo-hydrates in the liver—fat and glycogen? And does the hemoglobine—the coloring matter of the blood—subserve a like important part in nutrition?

Some fine figures are introduced to illustrate the histology of the liver, according to the German school; but nothing is said of the investigations of Beale, in England, who finds the liver cells contained in tubuli analogous to the kidney and other tubular glands. Thus we have but two forms of glands in the body: (1) the follicular, as the peptic and sebaceous, or these compounded into the racemose glands, as the lungs, meibomean glands, etc.; and (2) the tubular glands, as the sudoriparous and cerumenous, or these compounded into the kidney, the liver and the like.

The German school would make a *third* form of gland, in which the secreting cells are not contained in any envelope, but located upon the minute ramifications of the ducts; which absorb the secretion as manufactured, and thus carry it away. In this class they would include the liver, pancreas and salivary glands.

In the chapter upon "The Blood," the description of the spectra of venous and arterial blood, and the amœboid movements of the white blood globules, are the noticeable changes.

The subject of "Animal Heat" is much more fully discussed than in previous editions.

In describing the "Action of the Heart," the statement in former editions is repeated, viz.: that the ventricles elongate during contraction; but the figure of the frog's heart held between the thumb and finger in order to illustrate the action, is here omitted. Perhaps in subsequent editions the author will describe the shortening of the ventricles, with the majority of other observers.

Figures illustrating the tracings of the sphygmograph in health and disease, are the noticeable additions in the chapter on "The Circulation."

There are one hundred pages more in this edition relating to the nervous system than in the fourth. While the arrangement is nearly the same, there is a detail in description and treatment of each topic, which is much more satisfactory.

In the discussion of "The Function of the Cerebellum," we miss the figure of the pigeon, which in other editions so graphically illustrated the effect upon the movements of the bird—of the removal of the cerebellum, and which contrasted suggestively with that which is retained, showing the effects of the removal of the hemispheres.

The deep origin and distribution of the cranial nerves are fully described and finely illustrated. We must except, however, the illustration of facial paralysis, which wants the clearness and sharpness which have made the other editions, as well as this, in the main so attractive.

The section on "Reproduction" shows less improvement than any other part of the work, for the very good reason, doubtless, that it was here less needed.

During the past few years several new works on Physiology, and new editions of old works, have appeared, competing for the favor of the medical student; but none will rival this new edition of Dalton. As now enlarged, it will be found also to be, in general, a satisfactory work of reference for the practitioner. D. T. N.

